# Artemisinin Alleviates Cerebral Ischemia/Reperfusion-Induced Oxidative Damage via Regulating PHB2-Mediated Autophagy in the Human Neuroblastoma SH-SY5Y Cell Line

**DOI:** 10.1155/2022/6568748

**Published:** 2022-12-15

**Authors:** Menghan Jiang, Xiaoyi Lai, Yongjiang Zhang, Mengmeng Shen, Hongxia Ma, Anran Liu, Jiannan Wu, Junqiang Yan

**Affiliations:** ^1^Department of Neurology, The First Affiliated Hospital, College of Clinical Medicine of Henan University of Science and Technology, Luoyang 471003, China; ^2^Neuromolecular Biology Laboratory, The First Affiliated Hospital, College of Clinical Medicine of Henan University of Science and Technology, Luoyang 471003, China

## Abstract

Oxidative stress plays a key role in cerebral ischemia/reperfusion injury. Artemisinin (ART) has antioxidative stress activity in addition to its powerful antimalarial effects. In this article, we investigated the effect of ART on OGD/R-induced oxidative stress injury and its underlying mechanisms. We used oxygen-glucose deprivation/reoxygenation (OGD/R) to establish an in vitro model of cerebral ischemia/reperfusion (I/R) injury. CCK-8 and lactate dehydrogenase (LDH) release were used to assess cellular damage. Measurement of reactive oxygen species (ROS), malondialdehyde (MDA), superoxide dismutase (SOD), glutathione (GSH), and mitochondrial membrane potential (MMP) estimates oxidative stress-induced damage and protection from ART effect. OGD/R treatment aggravated oxidative stress damage, whereas ART reversed the effects of OGD/R. Autophagy is closely related to oxidative stress; in order to confirm whether the antioxidative stress effect of ART is related to PHB2-mediated autophagy, we examined the protein expression of prohibitin 2 (PHB2), TOMM20, p62, and the conversion of microtubule-associated protein light chain 3I (LC3I) to LC3II and found that the protein expression of PHB2, TOMM20, p62, and LC3II/LC3I was significantly correlated with OGD/R treatment. The colocalization of PHB2 and LC3, TOMM20, and LC3 was reduced after OGD/R treatment, and ART reversed this change. After silencing PHB2, the protective effect of ART against OGD/R-induced oxidative stress injury was reduced, the protein expressions of PHB2, TOMM20 and LC3II/LC3I and the colocalization of PHB2 and LC3, TOMM20, and LC3 were decreased. We used chloroquine to block the lysosomal pathway and found that ART increased the conversion of LC3I to LC3II, silencing PHB2 which inhibited the conversion of LC3I to LC3II, and impaired mitophagy. Our findings showed that ART attenuated OGD/R-induced oxidative stress damage through PHB2-mediated mitophagy. To the current knowledge, our study is the first to demonstrate that ART attenuates OGD/R-induced oxidative stress injury through PHB2-mediated autophagy in the human neuroblastoma SH-SY5Y cell line, which provided new insights into the treatment of OGD/R injury.

## 1. Introduction

Oxidative stress is the main cause of cerebral ischemia/reperfusion injury [1]. Free radicals that cerebral ischemia/reperfusion injury generated can directly damage mitochondria, and the damaged mitochondria produce a large number of free radicals [2], which is a vicious circle that aggravates the oxidative stress damage and eventually leading to nerve cells death [3, 4]. Therefore, reducing oxidative stress injury plays an important role in the treatment of stroke.

The impairment of autophagy exacerbates ischemic brain injury. A variety of autophagy receptors have been reported, including OPTN, DNP52, FUNDC1, and BNIP3. These receptors are localized or recruited to the mitochondrial outer membrane and then bind to LC3II to mediate mitophagy [5]. However, there are few studies on mitochondrial inner membrane autophagy receptors. Prohibitin 2 (PHB2) is an autophagy receptor on the mitochondrial inner membrane which binds to LC3 after the outer membrane of mitochondria rupture and then leads to mitophagy [6]. PHB2 could alleviate NLRP3-induced inflammation by improving mitophagy in renal tubular epithelial cells [7]. The mitochondria-targeted antioxidant mitoquinone could activate mitophagy through the PHB2 pathway and inhibit oxidative stress-related neuronal death [8]. PHB2-mediated mitophagy has become a topic of great interest in recent years, but its role in OGD/R-induced oxidative stress injury has not yet been reported.

Artemisinin (ART) is a sesquiterpene lactone extracted from the plant Artemisia annua. It is considered to be one of the most effective drugs for the treatment of malaria and has been used worldwide [9]. In recent years, more and more studies have proved that ART also has powerful antioxidative stress effect [10]. ART can alleviate the oxidative damage to SH-SY5Y cells and hippocampal neurons induced by hydrogen peroxide (H2O2) [11]. It has also been reported that ART can inhibit the death of PC12 cells and primary cerebral cortical neurons induced by sodium nitroprusside through ERK-mediated antioxidant stress [12]. Another study reported that ART can inhibit oxidative stress and inflammatory responses by activating Nrf2 and ROS-dependent p38 MAPK [13]. Our previous study found that ART could alleviate oxidative stress and apoptosis by inhibiting autophagy in MPP(+)-treated SH-SY5Y cells [14]. In the present study, we investigated whether ART could protect against OGD/R-induced SH-SY5Y cell injury by antioxidative stress and explored its molecular mechanism.

## 2. Materials and Methods

### 2.1. Cell Culture and Drug Treatment

Human neuroblastoma cell line SH-SY5Y from Sun Yat-sen University (Guangzhou, China) was cultured in DMEM/H (HyClone, Logan, UT, USA) containing 10% fetal bovine serum (Gibco, Grand Island, NY, USA) and 1% glutamine in an incubator at 37°C with 5% CO_2_. The medium was replaced every three days, and subculturing was performed when the cell density reached 80%. ART was purchased from Nanjing Dausf Biotechnology Co., Ltd. (DASF, Nanjing, China).

### 2.2. Oxygen-Glucose Deprivation and Reperfusion (OGD/R) Model

SH-SY5Y cells were cultured in glucose-free DMEM/H in a modularized anoxic incubator (Billups-Rothenberg, Del Mar, CA, USA) with 95% N2 and 5% CO_2_ at 37°C for 6 h for OGD. Then, the cells were cultured in normal medium under normoxic conditions (95% air, 5% CO_2_) at 37°C for another 24 h for reperfusion.

### 2.3. Establishment of Cell Line Stably Expressing PHB2-shRNA

PHB2-shRNA lentiviral particles were used to transfect SH-SY5Y cells to establish a cell line stably expressing PHB2-shRNA.Well-grown SH-SY5Y cells were inoculated in 12-well plates overnight. When the cell confluence reached 50%, 1 ml complete medium containing PHB2-shRNA lentiviral particles and polybrene (5 *μ*g/ml) were added to the culture plate and incubated overnight, which simultaneously transfected negative control shRNA lentiviral particles (NC shRNA) as a control. Remove the culture medium and replace with 1 ml complete medium without polybrene. Incubate the cells overnight. Select stably clone expressing PHB2-shRNA via puromycin dihydrochloride and replace medium with fresh puromycin-containing every 3–4 days until resistant colonies could be identified. The puromycin-resistant colonies were expanded, and transfection efficiency was detected using western blot. The cells stably expressing PHB2-shRNA were used in subsequent experiments.

### 2.4. Determination of OGD/R Time and ART Concentration

In order to observe the damage of OGD/R to cells at different time points, SH-SY5Y cells were divided into the control group and different time OGD/R groups (1, 2, 4, 6, and 8 h), and the cell viability, LDH release rate, cell morphology, and apoptosis rate were measured. In order to observe the toxicity of different concentrations of ART, cells were treated with different concentrations of ART (2.5, 5, 10, 20, and 40 *μ*m) for 24 h, and the cell viability was detected. To determine the optimal protective concentration of ART in the OGD/R model, cells were treated with OGD followed by ART for 24 hours during reperfusion, and the cell viability and LDH release rate were measured.

### 2.5. Cell Viability Assay

Cell viability was assessed using CCK-8 kits (SolarBio, CA1210, China). In brief, cells were seeded in 96-well plates (8 × 10^3^ cells/well) for overnight, and 100 *μ*l of medium containing 10 mM CCK-8 was added to each well. After incubating for 2 hours at 37°C, the absorbance at 450 nm was measured using a multimode microplate reader (Enspire, PerkinElmer, Singapore). Calculate cell viability according to the formula in the manual.

### 2.6. Apoptosis Assay

Apoptosis was assessed using Annexin V-PE/7AAD kit (SolarBio, CA1030, China). According to the manufacturer's instructions, dilute the binding buffer 1 : 4 with deionized water, then wash the cells twice with 4°C precooled PBS, resuspend the cells with 250 *μ*l binding buffer, and adjust the concentration to1 × 10^6^/ml; 5 *μ*l Annexin V/PE and 10 *μ*l 7AAD solution were added to 100 *μ*l cell suspension in a 5 ml flow tube, and the mixture was incubated in the dark at room temperature for 15 min and then added 400 *μ*l PBS to the reaction tube and analyzed by FACS.

### 2.7. LDH Release Rate Assay

The release rate of LDH was determined by Beyotime Lactate Dehydrogenase Cytotoxicity Detection Kit (C0017). According to the manufacturer's instructions, it inoculated SH-SY5Y cells into a 96-well culture plate and grouped them according to the instructions. When the cells grew to 80% full, add the drug to continue the culture. 1 h before the detection time point, add the LDH release reagent to the “sample maximum enzyme activity control well,” repeatedly pipette to mix, and continue to incubate. After reaching the predetermined time, the solution centrifuged at 400 g for 5 min to collect the supernatant to be tested. It was added with 60 *μ*l LDH detection solution to the remaining wells, mixed well, incubated in the dark at room temperature for 30 min, and measured the absorbance at 490 nm and 600 nm (reference wavelength). The LDH release rate was calculated according to the formula: LDH release rate (%) = (absorbance of processed sample − absorbance of sample control hole)/(absorbance of cell maximum enzyme activity − absorbance of sample control hole) × 100.

### 2.8. Intracellular ROS Detection

The ROS level was detected using the Beyotime reactive oxygen detection kit (S0033M) according to the manufacturer's instructions. The fluorescent probe (DCFH-DA) was diluted with serum-free culture solution at 1 : 1000 to a final concentration of 10 *μ*mol/L. The diluted DCFH-DA was added to an appropriate volume and incubated at 37°C for 20 min after the culture medium was removed. The cells were washed with serum-free culture solution three times to remove the DCFH-DA. The fluorescence intensity was calculated using an inverted microscope.

### 2.9. ROS Produced in the Mitochondria (mtROS) Detection

mtROS was measured by MitoSOX Red mitochondrial superoxide indicator (Abclonal, RM02822, Wuhan, China). After returning it to room temperature, it was added with 13 *μ*L DMSO to 50 *μ*g MitoSOX Red mitochondrial superoxide indicator and mixed well to prepare 5 mM storage solution. Before using, dilute it with PBS to 5 *μ*M working solution and then add the suitable working solution to cover the cell slide. After incubating the slides for 10 min at 37°C in the dark, wash them for three times with PBS. The cells were counterstained with DAPI and observed under a fluorescence microscope.

### 2.10. MDA Assay

After treatment, the cells were lysed and centrifuged at 10,000g for 10 min to collect the supernatant. The MDA was detected using Beyotime Lipid Oxidation (MDA) Detection Kits (S0131M), according to the manufacturer's instructions. The absorbance was measured at 450 nm (reference wavelength) and 532 nm using a microplate analyzer. The absorbance reading at 532 nm minus the absorbance reading at 450 nm was taken to be the measured reading. The concentration of MDA was calculated according to the standard curve: MDA content = (concentration of MDA in the sample^∗^ sample volume)/protein mass.

### 2.11. GSH Assay

The cells were collected by centrifugation after washing with PBS, and the supernatant was absorbed. Glutathione was detected using glutathione detection kits (Beyotime s0052), according to the manufacturer's instructions. The absorbance of the standard substance and the samples was measured at 412 nm using a microplate analyzer. The concentration of GSH was calculated according to the standard curve: GSH content = (concentration of GSH in the sample^∗^ sample volume)/protein mass.

### 2.12. SOD Activity Detection

SOD can catalyze the disproportionation of superoxide anion to produce hydrogen peroxide (H_2_O_2_) and oxygen (O_2_). The total SOD activity was detected using Beyotime SOD detection kits (S0101M). According to the manufacturer's instructions, cells were washed once with precooled PBS, 200 *μ*L of sample preparation solution was added to 1 × 10^6^ cells, which were lysed, and the solution was centrifuged at 4°C at 12,000g for 5 min to collect the supernatant. The absorbance of the sample at 450 nm and 650 nm (reference wavelength) was measured using a microplate analyzer, and the absorbance reading at 450 nm minus the absorbance reading at the reference wavelength was taken as the measured reading. SOD enzyme activity = SOD enzyme activity unit in the sample/protein mass.

### 2.13. Mitochondrial Membrane Potential (MMP) Assay

The changes of MMP were detected using mitochondrial membrane potential assay kit with JC-1 (Beyotime Biotechnology, C2006, China). According to the manufacturer's instructions, 1 ml of cell culture solution or 1 ml of JC-1 staining working solution was added to 1 × 10^6^ cells after washing the cells with PBS, and the mixture was incubated at 37°C for 20 min after thorough mixing. The supernatant was aspirated and washed twice with JC-1 staining buffer, and then the appropriate amount of cell culture solution was added and observed under an inverted fluorescence microscope. JC-1 monomer (green fluorescence) was detected in the GFP channel, and JC-1 polymer (red fluorescence) was detected in the TRITC channel.

### 2.14. Western Blot Assay

Cell lysates were prepared by incubating cells in a RIPA lysis buffer containing 1% PMSF (CWBIO, CW2333S, China) on ice for 30 min. The protein concentrations were determined using BCA Protein Assay Kits (CWBIO, CW2011S, China). Protein samples were loaded onto SDS-PAGE gels and run for 45 min, and protein samples were transferred to PVDF membranes and sealed with 5% skim milk in TBST for 1 h at room temperature. The membrane was then incubated overnight with the primary antibodies at 4°C. The primary antibodies used were as follows: Parkin (14060-1-AP, 1 : 1000, Proteintech), prohibitin 2 (12295-1-AP, 1 : 5000, Proteintech), LC3(14600-1-AP, 1 : 1000, Proteintech), p62 (66184-1-lg, 1 : 5000 Proteintech), and *β*-actin (CW0096M, 1 : 3000, CWBIO). After washing three times with PBST, HRP-conjugated goat anti-rabbit secondary antibody (1 : 50000, CWBIO) or goat anti-mouse secondary antibody (1 : 50000, CWBIO) was added, and the samples were then incubated at room temperature for 1 h. The images were obtained using universal hood electrophoretic imaging cabinet (Bio-Rad, USA) and analyzed using ImageJ.

### 2.15. Immunofluorescence Double Staining

The colocalization of PHB2/LC3 and TOMM20/LC3 was identified using immunofluorescence staining. The cell slides were fixed with 100% iced methanol for 5 min and washed three times with PBS for 5 min each time. The slides were then permeated with 0.25% Triton X-100 for 10 min and washed with PBS three times, for 5 min each time. After sealing with 1% BSA in PBST for 30 min, the cells were incubated overnight with PHB2 or TOMM20 antibody (anti-rabbit) and LC3 antibody (anti-mouse) at 4°C and then washed with PBS three times for 5 min each time. Goat anti-rabbit (Alexa Fluro555) and goat anti-mouse (Alexa Fluro488) fluorescent secondary antibodies were added at room temperature, and the mixture was incubated for 1 h in the dark before being washed with PBS three times for 5 min each time. After DAPI counterstaining and mounting, the colocalization of PHB2/LC3 and TOMM20/LC3 was imaged using a fluorescence microscope (Olympus, BX53; Olympus, U-RFL-T).

## 3. Results

### 3.1. Effect of OGD/R on SH-SY5Y Cells at Different Times

**Figure 1:**
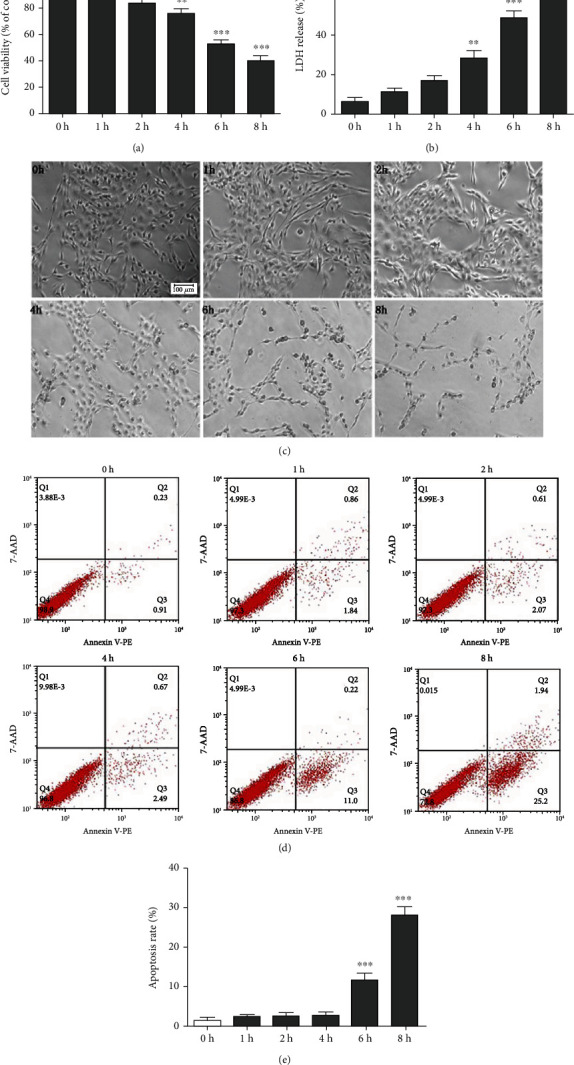
Cell viability at different times of OGD/R (a). LDH release at different times of OGD/R (b). Cell morphology at different times of OGD/R (c). Flow cytometry was used to detect the cell apoptosis at different times of OGD/R (d), and the cell apoptosis rate was analyzed (e). Values were expressed as mean ± SD (*n* = 3). Bar = 50 *μ*m. ^∗^*P* < 0.05 vs. 0 h group, ^∗∗^*P* < 0.01 vs. 0 h group, and ^∗∗∗^*P* < 0.001 vs. 0 h group.

CCK-8 results showed that the cell viability was significantly different from the control group from OGD/R 2 h which was 87.4% and decreased to 52.9% at OGD/R 6 h and to 38.7% at OGD/R 8 h (Figure 1(a)). The release of LDH increased in a time-dependent manner, and the release rate of LDH was significantly different from the control group from OGD/R 4 h which was 42.5% and increased to 48.8% at OGD/R 6 h and 67.8% at OGD/R 8 h (Figure 1(b)). We found that the cell morphology changed after OGD/R, the cells began to change round and shrank after OGD/R 4 h, and the cells died obviously after OGD/R 6 h which showed that the number of cells was significantly reduced (Figure 1(c)). The Annexin V-PE/7AAD results showed that the apoptosis rate was significantly increased from OGD/R 6 h compared with the control group (Figures 1(d) and 1(e)). Based on the above results, we chose OGD/R 6 h for the in vitro study of I/R in our research.

### 3.2. ART Increased Cell Viability and Reduced the Release of LDH in OGD/R-treated Cells

**Figure 2:**
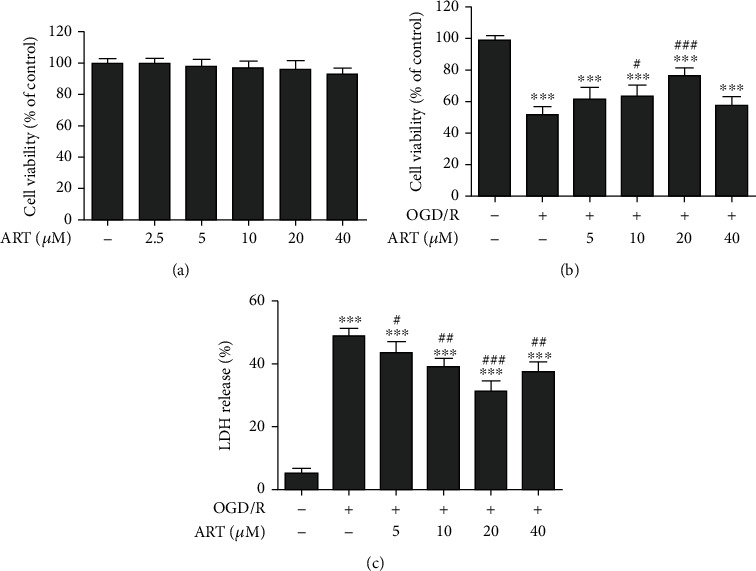
Effect of different concentrations of ART alone for 24 h on cell viability (a). Effect of ART for 24 h on cell viability after OGD/R for 6 h (b). Effect of ART for 24 h on LDH release after OGD/R for 6 h (c). Values were expressed as mean ± SD (*n* = 6). ^∗∗∗^*P* < 0.001 vs. control group, ^#^*P* < 0.05 vs. OGD/R group; ^##^*P* < 0.01 vs. OGD/R group, ^###^*P* < 0.001 vs. OGD/R group.

After treatment with different concentrations of ART (2.5, 5, 10, 20, and 40 *μ*M) for 24 h, no significant difference in cell viability was observed compared to the control group (Figure 2(a)). Then, we treated the cells with OGD/R and treated them with 5, 10, 20, or 40 *μ*M ART for 24 h during reperfusion. Compared with the OGD/R group, 10 *μ*M ART and 20 *μ*M ART treatment significantly improved the cell viability, but 20 *μ*M ART treatment had extremely significant differences (Figure 2(b)). In the study of LDH release, we found that compared with the OGD/R group, ART could significantly reduce the release of LDH from 5 *μ*M to 40 *μ*M, and when the concentration of ART was 20 *μ*M, there was an extremely significant difference (Figure 2(c)). Therefore, we selected 20 *μ*M ART for follow-up research [11, 15].

### 3.3. ART Alleviated OGD/R-Induced Oxidative Stress Injury

**Figure 3:**
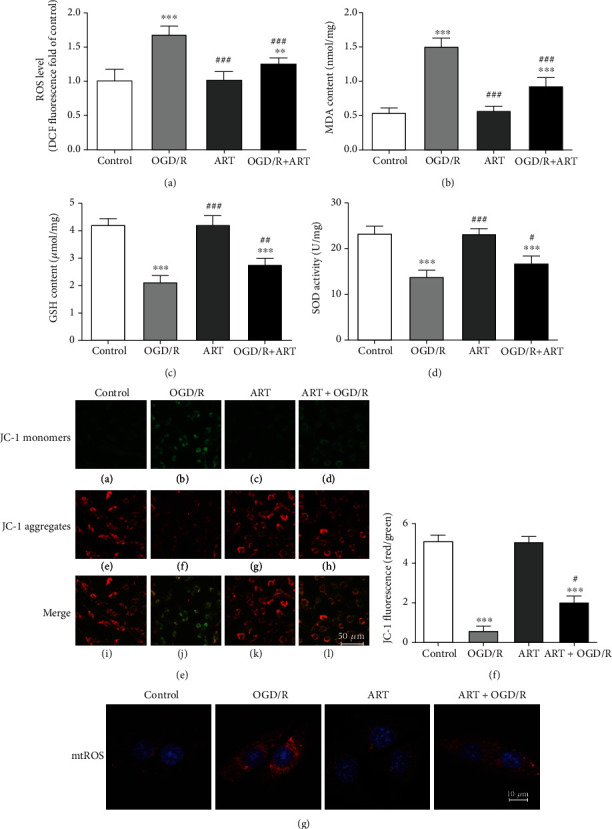
ART treatment decreased the OGD/R-induced ROS levels (a) and MDA content (b) and increased the GSH content (c) and SOD activity (d). ART alleviated the OGD/R-induced mitochondrial damage. ART alleviated OGD/R-induced depolarization of mitochondrial membrane potential (MMP). JC-1 staining was performed to assess MMP (e). Red fluorescence to green fluorescence was analyzed to evaluate the ratio of mitochondrial depolarization (f). ART treatment reduced the production of mtROS induced by OGD/R (g). Values were expressed as mean ± SD (*n* = 6). Bar = 50 *μ*m. ^∗∗^*P* < 0.01 vs. control group, ^∗∗∗^*P* < 0.001 vs. control group; ^#^*P* < 0.05 vs. OGD/R group, ^##^*P* < 0.01 vs. OGD/R group, and ^###^*P* < 0.001 vs. OGD/R group.

The effect of ART on OGD/R-induced oxidative stress injury was evaluated by detecting ROS, MDA, GSH levels, SOD activity, MMP, and mtROS.  Figure 3(a) indicates that ROS level in the OGD/R group significantly increased to 1.70 ± 0.10 (^∗∗∗^*P* < 0.001, *n* = 6), while it was only 1.01 ± 0.09 in the control group. ART significantly reduced the ROS level which was increased by OGD/R to 1.22 ± 0.08 (^###^*P* < 0.001, *n* = 6).

Figure 3(b) indicates that MDA content in the OGD/R group was significantly increased to 1.57 ± 0.16 nmol/mg which was only 0.5 ± 0.15 nmol/mg in the control group (^∗∗∗^*P* < 0.001, *n* = 6). ART reduced the MDA content which was increased by OGD/R to 0.97 ± 0.14 nmol/mg (^###^*P* < 0.001, *n* = 6).

Figure 3(c) indicates that GSH content of the OGD/R group was significantly decreased to 4.57 ± 0.40 *μ*mol/mg (^∗∗∗^*P* < 0.001, *n* = 6) which was 1.76 ± 0.25 *μ*mol/mg in the control group. ART significantly increased the GSH content which was decreased by OGD/R to 3.55 ± 0.48 nmol/mg (^##^*P* < 0.01, *n* = 6).

Figure 3(d) indicates that SOD activity in OGD/R-treated cells was significantly decreased to 11.50 ± 1.50 U/mg (^∗∗∗^*P* < 0.001, *n* = 6) which was 22.83 ± 2.02 U/mg in the control group. ART significantly increased the SOD activity which was decreased by OGD/R to 18.33 ± 1.53 (^#^*P* < 0.05, *n* = 6). However, the effect of ART treatment alone on the level of ROS, MDA, GSH, and SOD activity was not significantly different from the control group (*P* > 0.05, *n* = 6).

Depolarization of MMP is a hallmark event in the early stages of apoptosis. To investigate whether ART had a protective effect on OGD/R-induced mitochondrial damage in SH-SY5Y cells, we used mitochondrial membrane potential assay kit with JC-1 to assay the changes of MMP (Figures 3(e) and 3(f)). Green fluorescence indicates JC-1 monomer, indicating lower MPP, and red fluorescence represents JC-1 aggregate, indicating higher MPP. The relative ratio of red/green fluorescence is usually used to measure the proportion of mitochondrial depolarization. As shown in Figure 3(e), in the control group and the ART group, JC-1 staining showed bright red fluorescence and extremely weak green fluorescence, suggesting that the MMP was in a normal state. After OGD/R, the red fluorescence significantly decreased while the green fluorescence significantly increased, suggesting the depolarization of MMP. ART alleviated the OGD/R-induced depolarization of MMP.  Figure 3(f) shows the ratio of red/green fluorescence intensity.

As with the results of ROS test, we also found that mtROS levels of the OGD/R group increased significantly compared to the control group, and ART treatment significantly decreased the OGD/R-induced mtROS level (Figure 3(g)).

### 3.4. ART Reversed the Decrease of Autophagy Level induced by OGD/R

**Figure 4:**
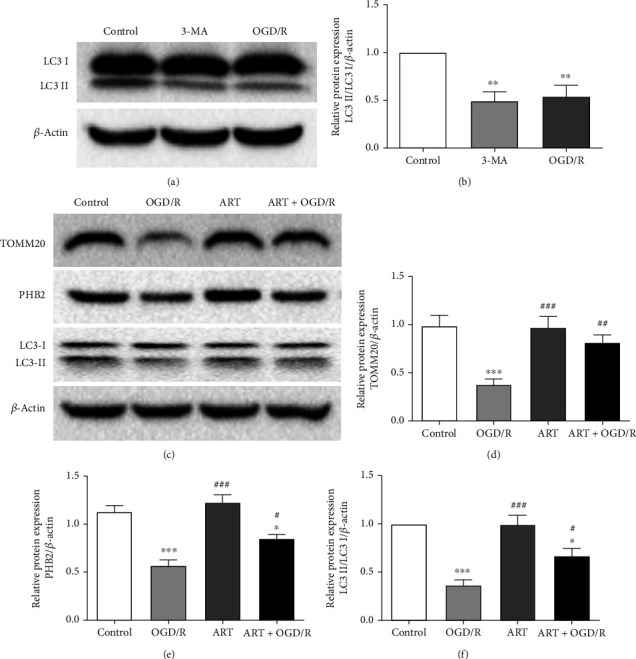
SH-SY5Y cells were treated with 3-MA or OGD/R, and the protein expression of LC3II/LC3I was detected by western-blot (a). The quantitative analysis of the protein expression of LC3II/LC3I (b). SH-SY5Y cells were treated with ART for 24 hours under normal conditions after 6h of OGD treatment. The protein expression of TOMM20, PHB2, and LC3II/LC3I was detected by western blot (c). The quantitative analysis of the protein expression of TOMM20, PHB2, and LC3II/LC3I (d-f). Values were expressed as mean ± SD (*n* = 3). ^∗^*P* < 0.05 vs. control group, ^∗∗^*P* < 0.01 vs. control group, and ^∗∗∗^*P* < 0.001 vs. control group; ^#^*P* < 0.05 vs. OGD/R group, ^##^*P* < 0.01 vs. OGD/R group, and ^###^*P* < 0.001 vs. OGD/R group.

To prove the effect of OGD/R on autophagy, we treated the cells with 3-MA and OGD/R respectively. Compared with the control group, both OGD/R and 3-MA treatment could significantly reduce the expression of LC3II/LC3I. However, there was no significant difference in the expression of LC3II/LC3I between the OGD/R group and 3-MA group (Figures 4(a) and 4(b)).

To further investigate whether autophagy is involved in the molecular mechanism of ART protection, we used western blot to detect the protein expression of TOMM20, PHB2, and LC3II/LC3I (Figures 4(c)–4(f)). Compared with the control group, OGD/R treatment significantly reduced the protein expression of TOMM20 and PHB2 and the ratio of LC3II/LC3I. Compared with the OGD/R group, ART significantly reversed OGD/R-induced reduction of TOMM20, PHB2, and LC3II/LC3I levels. However, ART treatment alone had no significant effect on the expression of TOMM20, PHB2, and LC3II/LC3I compared with the control group.

In addition, we detected the protein expression of p62 (supplementary Fig. 1). Compared with the control group, OGD/R treatment significantly increased the protein expression of p62. Compared with the OGD/R group, ART reversed OGD/R-induced increase of p62.

### 3.5. Silencing PHB2 Eliminated the Protection of ART against OGD/R-Induced Oxidative Stress Damage

**Figure 5:**
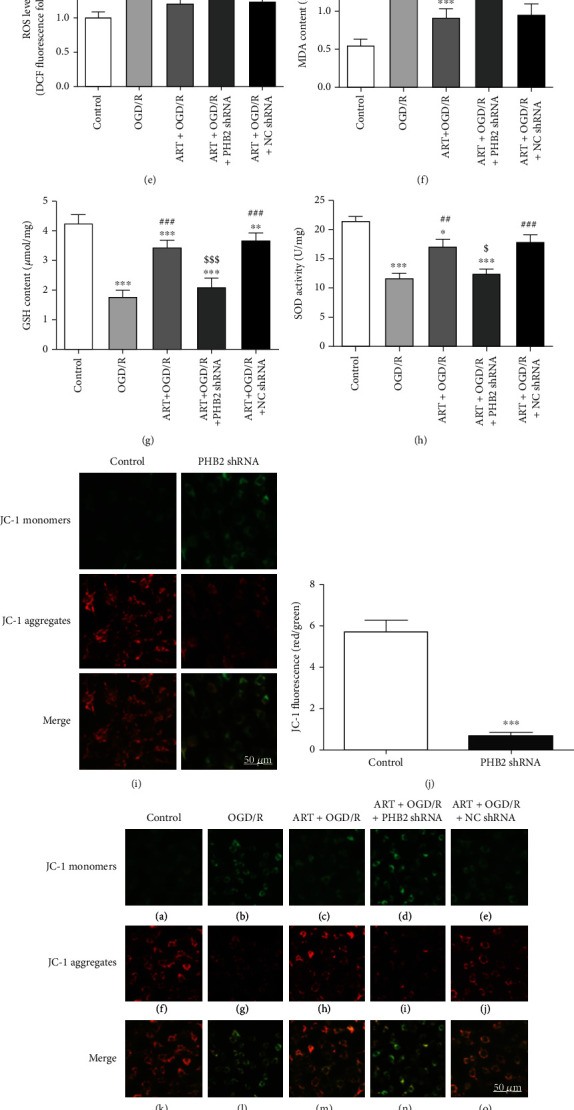
Silencing PHB2 caused oxidative stress damage (a–d) and leaded to depolarization of MMP (i, j). Silencing PHB2 eliminated the protection of ART against OGD/R-induced oxidative stress in SH-SY5Y cells (e–h) and eliminated the protection of MMP (k, l). Silencing PHB2 eliminated the protective effect of ART on the viability of SH-SY5Y cells (m). Values were expressed as mean ± SD (*n* = 6). Bar = 50 *μ*m. ^∗^*P* < 0.05 vs. control group, ^∗∗^*P* < 0.01 vs. control group, and ^∗∗∗^*P* < 0.001 vs. control group; ^##^*P* < 0.01 vs. OGD/R group, ^###^*P* < 0.001 vs. OGD/R group; ^$^*P* < 0.05 vs. ART+OGD/R group, ^$$^*P* < 0.001 vs. ART+OGD/R group, and ^$$$^*P* < 0.001 vs. ART+OGD/R group.

To demonstrate whether silencing PHB2 had an effect on oxidative stress and MMP, we examined the levels of ROS, MDA, GSH, SOD activity, and MMP after silencing PHB2. Compared with the control group, the ROS level and MDA content in the PHB2 shRNA group were significantly increased, while the GSH content and SOD activity in the PHB2 shRNA group were significantly decreased (Figures 5(a)–5(d)). In the control group, JC-1 staining showed bright red fluorescence and weak green fluorescence, suggesting higher MMP. After silencing PHB2, red fluorescence was significantly reduced, and green fluorescence was significantly increased, indicating MMP depolarization (Figures 5(i) and 5(j)).

To investigate whether PHB2 mediates the protective effect of ART against OGD/R-induced oxidative stress injury, PHB2-silenced SH-SY5Y cells were treated with OGD/R and ART, and then ROS, MDA, GSH, SOD, and MMP were detected.  Figure 5(e) indicates that ROS level in the OGD/R-treated group significantly increased to 1.67 ± 0.09 (^∗∗∗^*P* < 0.001, *n* = 6), while it was only 1.03 ± 0.08 in the control group. ART significantly reduced the ROS level induced by OGD/R to 1.20 ± 0.07 (^##^*P* < 0.01, *n* = 6). The ROS level in the ART+OGD/R+PHB2 shRNA group was 1.69 ± 0.07 which was not significantly different from the OGD/R group (*P* > 0.05, *n* = 6). The ROS level in the ART+OGD/R+NC shRNA group was 1.24 ± 0.14 which was significantly decreased than that in the OGD/R group (^##^*P* < 0.01, *n* = 6). However, the ROS level of the ART+OGD/R+NC shRNA group was not significantly different from that of the ART+OGD/R group (*P* > 0.05, *n* = 6).

Figure 5(f) indicates that MDA content in the control group was 0.55 ± 0.11 nmol/mg which was significantly increased to 1.63 ± 0.15 nmol/mg in the OGD/R group (^∗∗∗^*P* < 0.001, *n* = 6). ART significantly reduced the MDA content which was increased by OGD/R to 0.87 ± 0.12 nmol/mg (^###^*P* < 0.001, *n* = 6). The MDA content in the ART+OGD/R+PHB2 shRNA group was 1.43 ± 0.16 nmol/mg which was significantly increased than that in the ART+OGD/R group (^&&^*P* < 0.01, *n* = 6). The MDA content in the ART+OGD/R+NC shRNA group was 0.91 ± 0.09 nmol/mg, which was significantly different lower than that in the OGD/R group (^###^*P* < 0.001, *n* = 6) but not significantly different from the ART+OGD/R group (*P* > 0.05, *n* = 6).

Figure 5(g) indicates that GSH content in the control group was 4.43 ± 0.42 *μ*mol/mg, while it significantly reduced to 1.80 ± 0.15 *μ*mol/mg in the OGD/R group (^∗∗∗^*P* < 0.001, *n* = 6). ART significantly increased GSH content which was decreased by OGD/R to 3.48 ± 0.26 *μ*mol/mg (^###^*P* < 0.001, *n* = 6). GSH content in the ART+OGD/R+PHB2 shRNA group was 2.11 ± 0.34 *μ*mol/mg, which was not significantly different from the OGD/R group (*P* > 0.05, *n* = 6). However, there was a significant difference in GSH content between the ART+OGD/R+PHB2 shRNA group and ART+OGD/R group (^&&&^*P* < 0.001, *n* = 6). GSH content in the ART+OGD/R+NC group was 3.68 ± 0.35 *μ*mol/mg, which was significantly different from the OGD/R group (^###^*P* < 0.001, *n* = 6). However, the GSH between the ART+OGD/R+NC group and the ART+OGD/R group was no significantly different (*P* > 0.05, *n* = 6).

Figure 5(h) indicates that compared with the control group, SOD activity in the OGD/R group decreased from 21.83 ± 2.02 U/mg to 11.17 ± 1.61 U/mg (^∗∗∗^*P* < 0.001, *n* = 6). ART significantly increased the SOD activity which was increased by OGD/R to 18.0 ± 2.18 U/mg (^##^*P* < 0.01, *n* = 6), which was not significantly different compared with the control group (*P* > 0.05, *n* = 6). SOD activity in the ART+OGD/R+PHB2 shRNA group was 12.47 ± 1.50 U/mg, which was not significantly different from the OGD/R group (*P* > 0.05, *n* = 6), but was significantly different from the ART+OGD/R group (^&^*P* < 0.05, *n* = 6). SOD activity in the ART+OGD/R+NC shRNA group was 17.27 ± 1.10 U/mg, which was significantly increased than that in the OGD/R group (^#^*P* < 0.05, *n* = 6).

JC-1 staining showed bright red fluorescence and weak green fluorescence in the control group (Figure 5(k)), which suggested that the MMP was in a normal state. After OGD/R, the red fluorescence was extremely weak, while the green fluorescence was enhanced, suggesting the depolarization of MMP. ART alleviated the OGD/R-induced depolarization of MMP, i.e., green fluorescence was darker than OGD/R, and red fluorescence was stronger than OGD/R. After silencing of PHB2, ART-alleviated OGD/R-induced depolarization of MMP disappeared. These results suggested that ART could not exert its protection effect against OGD/R-induced mitochondria damage after PHB2 silencing.  Figure 5(l) shows the ratio of red/green fluorescence intensity.

To investigate the effects of silencing PHB2 on cell viability upon OGD/R or ART treatment, we detected the cell viability using CCK-8 kits (Figure 5(m)). Compared with the control group, the cell viability in the OGD/R group was significantly reduced (^∗∗∗^*P* < 0.001, *n* = 6), and ART significantly increased the cell viability which decreased by OGD/R (^##^*P* < 0.01, *n* = 6). The cell viability in the ART+OGD/R+PHB2 shRNA group was not significantly different from the OGD/R group (*P* > 0.05, *n* = 6) but was significantly lower than the ART+OGD/R group (^&&&^*P* < 0.001, *n* = 6). The cell viability in the ART+OGD/R+NC shRNA group was significantly higher than the OGD/R group (^##^*P* < 0.01, *n* = 6) but was not different from the ART+OGD/R group (*P* > 0.05, *n* = 6).

### 3.6. Silencing PHB2 Reversed the Increase of Autophagy Caused by ART in the OGD/R Model

**Figure 6:**
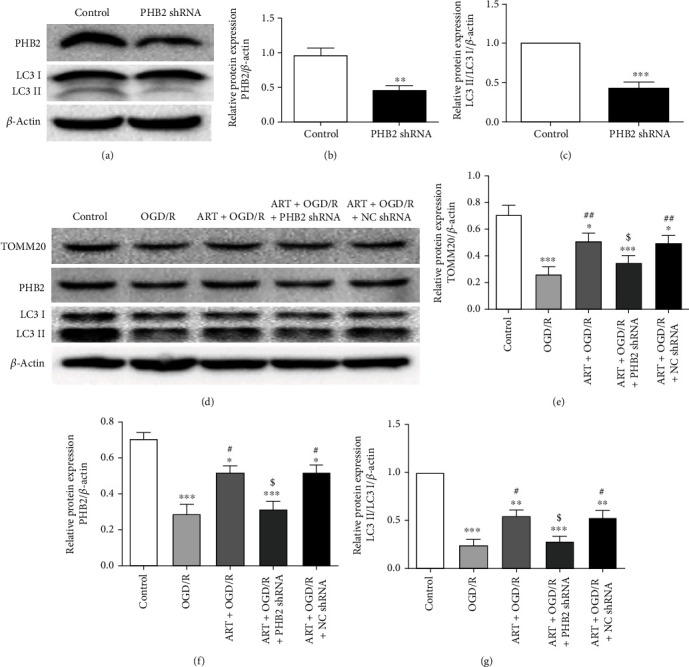
The protein expression of PHB2 and LC3II/LC3I was detected by western blot after silencing PHB2 (a), and the protein expression was quantitatively analyzed (b, c). Cells were treated with ART or OGD/R after silencing PHB2, the protein expression of TOMM20, PHB2, and LC3II/LC3I was detected by western blot (d), and the protein expression of TOMM20, PHB2, and LC3II/LC3I was quantitatively analyzed (e–g). Values were expressed as mean ± SD (*n* = 3). ^∗^*P* < 0.05 vs. control group, ^∗∗^*P* < 0.01 vs. control group, and ^∗∗∗^*P* < 0.001 vs. control group; ^#^*P* < 0.05 vs. OGD/R group, ^##^*P* < 0.05 vs. OGD/R group.

In order to verify the knockout efficiency of PHB2, we tested the expression of PHB2 and LC3II/LC3I after PHB2-shRNA lentiviral particles were transfected into SH-SY5Y cells (Figures 6(a)–6(c)). Compared with the control group, the level of PHB2 and the ratio of LC3II/LC3I were significantly reduced in the PHB2 shRNA group.

In order to further explore whether PHB2 mediated the molecular mechanism of ART protection through autophagy, we used PHB2 silenced SH-SY5H cells to detect the protein expression of TOMM20, PHB2, and LC3II/LC3I after OGD/R treatment or ATR treatment (Figures 6(d)–6(g)). Compared with control group, OGD/R significantly reduced the levels of TOMM20, PHB2, and LC3II/LC3I. However, ART significantly reversed the OGD/R-induced reduction of TOMM20, PHB2, and LC3II/LC3I. Compared with the ART+OGD/R group, the expressions of TOMM20, PHB2, and LC3II/LC3I in the ART+OGD/R+PHB2 shRNA group were significantly reduced. No significant differences were found in the protein expression of TOMM20, PHB2, and LC3II/LC3I between ART+OGD/R+NC shRNA group and ART+OGD/R group.

### 3.7. Silencing PHB2 Reduced the Colocalization Expression of PHB2 and LC3, TOMM20, and LC3

**Figure 7:**
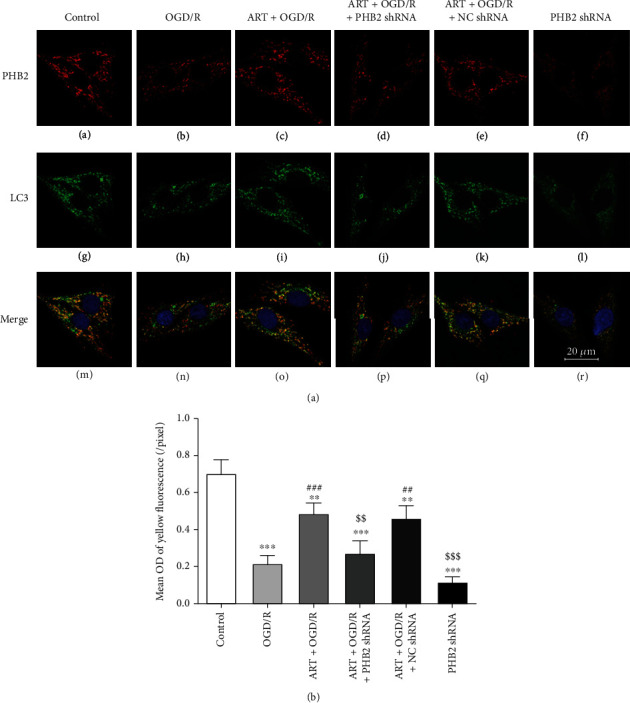
Double immunofluorescence staining was performed to observe the effect of silencing PHB2 on the colocalization expression of PHB2 and LC3. Red fluorescence represents the expression of PHB2 (a–f), green fluorescence represents the expression of LC3 (g–l), and yellow fluorescence represents the colocalization of PHB2 and LC3 (m–r). Bar = 20 *μ*m. ^∗∗^*P* < 0.01 vs. control group, ^∗∗∗^*P* < 0.001 vs. control group; ^##^*P* < 0.01 vs. OGD/R group, ^###^*P* < 0.001 vs. OGD/R group; ^$$^*P* < 0.01 vs. ART+OGD/R group, ^$$$^*P* < 0.001 vs. ART+OGD/R group.

To further verify whether PHB2 and LC3 interact, we used immunofluorescence double staining for colocalization staining of PHB2 and LC3 (Figure 7(a), A–R). We analyzed the yellow fluorescence intensity, which represents the coexpression of PHB2 and LC3 (Figure 7(b)). Compared with the control group, the colocalization of PHB2 and LC3 in the OGD/R group and PHB2 shRNA group was significantly decreased. The colocalization of PHB2 and LC3 in the ART+OGD/R group and ART+OGD/R+NC shRNA group was significantly increased compared with the OGD/R group. Compared with the ART+OGD/R group, the colocalization of PHB2 and LC3 in the ART+OGD/R+PHB2 shRNA group was significantly decreased.

Furthermore, we reached a similar conclusion by evaluating the colocalization degree of each merged images through Pearson's correlation coefficient (PCC). The PCC values of the control group, ART+OGD/R group, and ART+OGD/R+NC shRNA group were 0.53, 0.55, and 0.56, respectively, showing high colocalization. The PCC values of the OGD/R group, ART+OGD/R+PHB2 shRNA group, and PHB2 shRNA group were 0.34, 0.31, and 0.31, respectively, indicating lower colocalization (Supplementary Fig. 3).

We also investigate the colocalization of TOMM20 and LC3 and quantitatively analyzed the colocalization expression by PCC (Supplementary Fig. 4). Compared with the control group, the PCC value in the OGD/R group and PHB2 shRNA group was significantly decreased, indicating lower colocalization. Compared with the OGD/R group, the PCC values in the ART+OGD/R group and ART+OGD/R+NC shRNA group increased significantly, indicating high colocalization. Compared with the ART+OGD/R group, the PCC value in the ART+OGD/R+PHB2 shRNA group was also decreased.

### 3.8. ART Increased LC3II/LC3I Expression through PHB2

**Figure 8:**
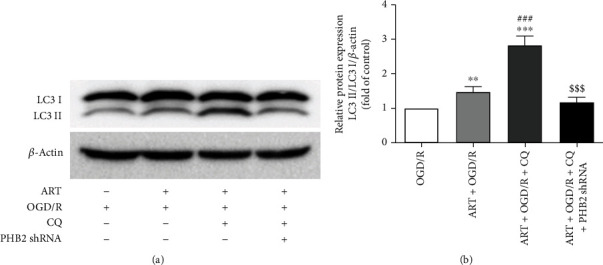
SH-SY5Y cells stably expressing PHB2-shRNA were treated with ART and CQ for 24 h after OGD treatment, and the LC3II/LC3I ratio was reduced. The protein expression of LC3II/LC3I was detected by western blot (a), and the protein expression wasquantitatively analyzed (n). Values were expressed as mean ± SD (*n* = 3). ^∗∗^*P* < 0.01 vs. OGD/R group, ^∗∗∗^*P* < 0.001 vs. OGD/R group; ^###^*P* < 0.001 vs. ART+OGD/R group; ^$$$^*P* < 0.001 vs. ART+OGD/R +CQ group.

We detected the LC3II/LC3I ratio and found that compared with the OGD/R group, the ratio of LC3II/LC3I in the ART+OGD/R group and ART+OGD/R+ chloroquine (CQ) group was significantly increased. Compared with the ART+OGD/R group, the ratio of LC3II/LC3I was significantly increased in the ART+OGD/R+CQ group. However, the ratio of LC3II/LC3I in ART+OGD/R+CQ+PHB2 shRNA was significantly decreased compared with the ART+OGD/R+CQ group (Figures 8(a) and 8(b)). These results indicated that ART enhanced mitophagy by increasing the conversion of LC3I to LC3II and binding to PHB2.

We examined LC3 and p62's expression in CQ-treated OGD/R condition to reveal the autophagic flux. Compared with the OGD/R group, the ratio of LC3II/LC3I in the OGD/R+CQ group was significantly increased. Compared with the OGD/R+CQ group, the ratio of LC3II/LC3I in the ART+OGD/R+CQ group was significantly increased, while the silence of PHB2 significantly reversed the increase (supplementary Fig. 2A-B). Compared with the OGD/R group, the p62 level in the OGD/R+CQ group was significantly increased, while ART significantly reversed the increase of p62 level in the OGD/R+CQ group. The p62 level in ART+OGD/R+CQ+PHB2 shRNA was significantly increased compared with the ART+OGD/R+CQ group (supplementary Fig. 2C-D).

## 4. Discussion

Oxidative stress is the main cause of I/R injury. The imbalance of oxidative and antioxidant processes leads to oxidative stress, which can lead to severe cellular necrosis and apoptosis. Increased production of reactive oxygen species (ROS) is the main cause of oxidative stress. The lipid-rich brain tissue reacts with ROS to generate hydrogen peroxide free radicals, leading to membrane lipid peroxidation and neuronal damage [16]. In addition, the brain has relatively low levels of endogenous antioxidants, making neurons particularly sensitive to oxidative stress [17].

We used OGD/R to simulate an in vitro model of stroke. The cells were deprived of glucose and hypoxia for 6 h and then restored to glucose and reoxygenated for 24 h, because the cell viability dropped to about 50% of the cells in the control group at this time point, which was convenient to observe the protective effect of the drug. Our results confirmed that ART significantly increased the levels of intracellular antioxidant SOD and GSH after OGD/R and decreased the amount of ROS (including mitochondrial ROS) and MDA. ART attenuated mitochondrial damage by improving the depolarization of OGD/R-induced MMP, which suggested that ART could protect OGD/R-induced mitochondrial damage.

Mitochondrial dysfunction is closely related to ageing [18, 19] and various central nervous system diseases such as PD [19]. Activating mitophagy may prevent disease progression by regulating mitochondrial mass, maintaining mitochondrial function, and ultimately improving the intracellular environment. The clearance dysfunction of damaged mitochondria is an important factor in aggravating brain injury [20, 21]. Timely clearance of damaged mitochondria is very important in the treatment of OGD/R injury. Wu et al. [22] showed that hydrogen exerted neuroprotective effects on OGD/R-injured neurons by protecting mitochondrial function, while 3-MA further aggravated the injury and inhibited the protection of hydrogen. Zhou et al. [23] demonstrated that mitophagy inhibited oxidative stress by upregulating Parkin protein, thereby alleviating focal I/R injury in rats.

PHB2 could stabilize the mitochondrial inner membrane protease PARL and prevented PARL from cleaving PGAM5, and the intact PGAM5 could stabilize PINK1 on the mitochondrial outer membrane and then recruit Parkin and other receptors to promote mitophagy [24]. We investigated whether PHB2-mediated autophagy can reduce OGD/R-induced oxidative stress injury. Our results showed that OGD/R treatment decreased the protein expression of TOMM20, PHB2, and LC3, the colocalization of PHB2 and LC3, and the colocalization of TOMM20 and LC3, and increased the protein expression of p62, which indicated that level of mitophagy decreased. However, ART can reverse the protein expression and increase the colocalization of PHB2 and LC3, TOMM20, and LC3. The silence of PHB2 prevented the protective effect of ART which reversed the level of mitophagy. These findings suggested that the protective effect of ART on OGD/R-induced oxidative stress injury was achieved through PHB2-mediated mitophagy.

To further confirm that the antioxidative stress effect of ART is achieved by promoting autophagy, we used chloroquine to inhibit the lysosomal degradation pathway of LC3II and found that the conversion of LC3I to LC3II was significantly increased, while the conversion of LC3I to LC3II was decreased after PHB2 silencing. This suggests that ART enhances autophagy by increasing the binding of PHB2 and LC3.

## 5. Conclusions

Our findings suggested that ART could reduce OGD/R-induced oxidative stress damage in SH-SY5Y cells, and the mechanism might be related to PHB2-mediated autophagy. To the current knowledge, our study is the first to demonstrate that ART attenuates OGD/R-induced oxidative stress injury through PHB2-mediated autophagy in the human neuroblastoma SH-SY5Y cell line, which provided new insights into the treatment of OGD/R injury.

## Data Availability

All data generated or analyzed during this study are included in this published article.

## Supplementary Materials

Supplementary Materials Supplementary Figure 1: the protein expression of p62 was detected by western blot (a). The quantitative analysis of protein expression of p62 (b). Values were expressed as mean ± SD (*n* = 3). ^∗^*P* < 0.05 vs. control group; ^##^*P* < 0.01 vs. OGD/R group; ^###^*P* < 0.001 vs. OGD/R group. Supplementary Figure 2: the protein expression of LC3II/LC3I (a) and p62 (c) was detected by western blot, and the protein expression was quantitatively analyzed (b and d). Values were expressed as mean ± SD (*n* = 3). ^∗∗^*P* < 0.01 vs. OGD/R group, ^∗∗∗^*P* < 0.001 vs. OGD/R group; ^&^*P* < 0.05 vs. OGD/R +CQ group, ^&&&^*P* < 0.001 vs. OGD/R +CQ group; ^$^*P* < 0.05 vs. ART+OGD/R +CQ group, ^$$$^*P* < 0.001 vs. ART+OGD/R +CQ group. Supplementary Figure 3: scatter plots of intensities of green and red pixels from the same spots in each fluorescent image. The red intensities are used as the *x*-coordinate and the green intensities as the *y*-coordinate. The frequencies of pixels appearing in the scatterplot were distinguished by colors, and the numbers beside the bar indicate the frequency level.
