# Supplementary material for: Artemisinin Alleviates Cerebral Ischemia/Reperfusion-Induced Oxidative Damage via Regulating PHB2-Mediated Autophagy in the Human Neuroblastoma SH-SY5Y Cell Line
Source: Oxid Med Cell Longev. 2022 Dec 15;2022:6568748. doi: 10.1155/2022/6568748 (PMC9780004; doi:10.1155/2022/6568748)
Supplement: Supplementary Materials — Supplementary Figure 1: the protein expression of p62 was detected by western blot (a). The quantitative analysis of protein expression of p62 (b). Values were expressed as mean ± SD (n = 3). ∗P < 0.05 vs. control group; ##P < 0.01 vs. OGD/R group; ###P < 0.001 vs. OGD/R group. Supplementary Figure 2: the protein expression of LC3II/LC3I (a) and p62 (c) was detected by western blot, and the protein expression was quantitatively analyzed (b and d). Values were expressed as mean ± SD (n = 3). ∗∗P < 0.01 vs. OGD/R group, ∗∗∗P < 0.001 vs. OGD/R group; &P < 0.05 vs. OGD/R +CQ group, &&&P < 0.001 vs. OGD/R +CQ group; $P < 0.05 vs. ART+OGD/R +CQ group, $$$P < 0.001 vs. ART+OGD/R +CQ group. Supplementary Figure 3: scatter plots of intensities of green and red pixels from the same spots in each fluorescent image. The red intensities are used as the x-coordinate and the green intensities as the y-coordinate. The frequencies of pixels appearing in the scatterplot were distinguished by colors, and the numbers beside the bar indicate the frequency level. [file 6568748.f1.docx]

Supplementary Materials

**Supplementary Figure 1.**

Supplementary Figure 1. The protein expression of p62 was detected by western-blot (A). The quantitative analysis of protein expression of p62 (B). Values were expressed as mean ± SD (n = 3). ^*^P < 0.05 vs. control group; ^##^P < 0.01 vs. OGD/R group; ^###^P < 0.001 vs. OGD/R group.

**Supplementary Figure 2.**

Supplementary Figure 2. The protein expression of LC3II/LC3I (A) and p62 (C) was detected by western-blot, the protein expression were quantitatively analyzed (B and D). Values were expressed as mean ± SD (n = 3). ^**^P < 0.01 vs. OGD/R group, ^***^P < 0.001 vs. OGD/R group; ^＆^P < 0.05 vs. OGD/R +CQ group, ^＆＆＆^P < 0.001 vs. OGD/R +CQ group; ^$^P < 0.05 vs. ART+OGD/R +CQ group, ^$$$^P < 0.001 vs. ART+OGD/R +CQ group.

**Supplementary Figure 3.**

Supplementary Figure 3. Scatter plots of intensities of green and red pixels from the same spots in each fluorescent image. The red intensities are used as the x-coordinate and the green intensities as the y-coordinate. The frequencies of pixels appearing in the scatterplot were distinguished by colors, and the numbers beside the bar indicate the frequency level.

**Supplementary Figure 4.**

Supplementary Figure 4. The co-localization of TOMM20 and LC3 were detected by immunofluorescence double staining (A), red fluorescence represents the expression of TOMM20, green fluorescence represents the expression of LC3, yellow fluorescence represents the co-localization of TOMM20 and LC3. The co-localization expression were quantitatively analyzed by Pearson's correlation coefficient (B). red intensities are used as the x-coordinate and the green intensities as the y-coordinate.
